# Model and Comparative Study for Flow of Viscoelastic Nanofluids with Cattaneo-Christov Double Diffusion

**DOI:** 10.1371/journal.pone.0168824

**Published:** 2017-01-03

**Authors:** Tasawar Hayat, Arsalan Aziz, Taseer Muhammad, Ahmed Alsaedi

**Affiliations:** 1 Department of Mathematics, Quaid-I-Azam University, Islamabad, Pakistan; 2 Nonlinear Analysis and Applied Mathematics (NAAM) Research Group, Department of Mathematics, Faculty of Science, King Abdulaziz University, Jeddah, Saudi Arabia; Tianjin University, CHINA

## Abstract

Here two classes of viscoelastic fluids have been analyzed in the presence of Cattaneo-Christov double diffusion expressions of heat and mass transfer. A linearly stretched sheet has been used to create the flow. Thermal and concentration diffusions are characterized firstly by introducing Cattaneo-Christov fluxes. Novel features regarding Brownian motion and thermophoresis are retained. The conversion of nonlinear partial differential system to nonlinear ordinary differential system has been taken into place by using suitable transformations. The resulting nonlinear systems have been solved via convergent approach. Graphs have been sketched in order to investigate how the velocity, temperature and concentration profiles are affected by distinct physical flow parameters. Numerical values of skin friction coefficient and heat and mass transfer rates at the wall are also computed and discussed. Our observations demonstrate that the temperature and concentration fields are decreasing functions of thermal and concentration relaxation parameters.

## 1. Introduction

There is a significant advancement in the nanotechnology due to its rich applications in the industrial and physiological processes. The modern researchers are engaged to explore the mechanisms through the nanomaterials. A solid-liquid mixture of tiny size nanoparticles and base liquid is known as nanofluid. The colloids of base liquid and nanoparticles have important physical characteristics which enhance their potential role in the applications of ceramics, drug delivery, paintings, coatings etc. Nanofluids are declared as super coolants because their heat absorption capacity is much higher than traditional liquids. The reduction of the system and enhancement in its performance can be achieved with the implications of nanoliquids. The term nanofluid was first introduced by Choi and Eastman [1] and they illustrated that the thermal properties of base liquids are enhanced when we add up the nanoparticles in it. Buongiorno [2] developed the model of nanoparticles by considering the thermophoretic and Brownian motion aspects. Further the recent developments on nanoliquids can be seen in the investigations [3–20].

The process of heat transfer occurs when there is a difference of temperature between the bodies or between the various parts of the same body. This process has vast technological and industrial use, for example, cooling of atomic reactors, power generation, energy production etc. The famous law of heat conduction proposed by Fourier [21] is mostly employed for heat transfer attributes since it appeared in the literature. Cattaneo [22] modified this law by including a term of relaxation time. This term overcomes the paradox of heat conduction. Christov [23] further modified the Cattaneo theory [22] by substituting the time derivative with Oldroyd upper-convected derivative. This theory is termed as the Cattaneo-Christov heat flux theory. Straughan [24] employed heat flux model by Cattaneo-Christov theory to explore thermal convection in horizontal layer of viscous liquid. Ciarletta and Straughan [25] showed the structural stability and uniqueness of solutions for an energy equation with heat flux by Cattaneo-Christov expression. Haddad [26] discussed the thermal instability in the Brinkman porous medium by employing heat flux with Cattaneo-Christov expression. Han et al. [27] addressed the stretched flow of Maxwell liquid through heat flux by Cattaneo-Christov expression. Mustafa [28] used heat flux through Cattaneo-Christov expression in order to explore heat transfer for flow of Maxwell material. He provided numeric and analytic solutions of governing flow systems. Khan et al. [29] provided a numerical analysis to study the thermal relaxation attributes in Maxwell material flow by an exponentially stretched surface. Recently, Hayat et al. [30] performed a comparative study for flows of viscoelastic materials by considering heat flux through Cattaneo-Christov expression.

At present the non-Newtonian materials have gained much attention because of their involvement in extensive industrial and engineering applications. Such applications involve bioengineering and polymeric liquids, plastics manufacturing, annealing and thinning of copper wires, food processing, petroleum production, drawing of stretching sheet through quiescent fluid and aerodynamic extrusion of plastic films etc. The well-known Navier-Stokes expression is not good enough to characterize the flows of non-Newtonian materials. A single relation is not sufficient to depict the characteristics of all the non-Newtonian materials. Therefore, different types of non-Newtonian relations are given in the literature. Amongst these relations, the elastico-viscous and second grade materials [31–36] are the simplest subclasses of differential type non-Newtonian materials which describe the effects of normal stress. Moreover the analysis of liquid-liquid two-phase flows are widely involved in several industrial processes such as spray processes, lubrication, natural gas networks, nuclear reactor cooling etc. Thus Gao et al. [37] provided a multivariate weighted complex network analysis to examine the nonlinear dynamic behavior in two-phase flow. Gao et al. [38] also addressed the multi-frequency complex network to explore the uncovering oil-water flow structure. Slug to churn flow transition by employing the multivariate pseudo Wigner distribution and multivariate multiscale entropy is reported by Gao et al. [39]. Gao et al. [40] provided a four-sector conductance method to explore the low-velocity oil-water two-phase flows. Recently Gao et al. [41] developed a novel multiscale limited penetrable horizontal visibility graph to analyze the nonlinear time series.

This research article presents a comparative study for Cattaneo-Christov double diffusion expressions in the boundary-layer flow of viscoelastic nanofluids by considering two classes of viscoelastic fluids. Constitutive relations for second grade and elastico-viscous fluids are considered. Brownian motion and thermophoresis aspects are considered. Most of the investigations in the literature are explained through the classical Fourier's and Fick's laws. The main purpose here is to utilize the generalized Fourier's and Fick's laws namely Cattaneo-Christov double diffusion expressions in the boundary-layer flow of viscoelastic nanofluids. Mathematical formulation of the present analysis is performed subject to both generalized Fourier's and Fick's laws namely Cattaneo-Christov double diffusion expressions. In particular the present study generalizes the results of ref. [35] by considering another model of elastico-viscous fluid, comparison and double diffusion of heat and mass transfer by Cattaneo-Christov theory. Thus to the best of the author's knowledge, no such attempt has been discussed in the literature yet. Transformation procedure is utilized to convert the partial differential system into the set of nonlinear ordinary differential system. The governing nonlinear system has been solved through the homotopy analysis method (HAM) [42–50]. Convergence of computed solutions is checked by plots and numerical data. The contributions of various pertinent parameters are studied and discussed. Heat and mass transfer rates at the surface are also analyzed through numerical values.

## 2. Formulation

Let us consider the steady two-dimensional (2D) flow of viscoelastic nanofluids over a linear stretching sheet with constant surface temperature and concentration. The flow models for elastico-viscous and second grade materials are considered. The Brownian motion and thermophoresis are taken into consideration. Here *x*–axis is along the stretching surface while *y*–axis is normal to the *x*–axis. The stretching velocity is *u*_*w*_(*x*) = *ax* with *a* > 0 as the constant. The heat and mass transfer mechanisms are examined through Cattaneo-Christov double diffusion expressions. Governing equations of mass, momentum, energy and nanoparticles concentration for boundary layer considerations are

$$\frac{\partial u}{\partial x}+\frac{\partial v}{\partial y}=0,$$ (1)

$$u\frac{\partial u}{\partial x}+v\frac{\partial u}{\partial y}=\nu \frac{\partial^{2}u}{\partial y^{2}}-k_{0}\left(u\frac{\partial^{3}u}{\partial x\partial y^{2}}+v\frac{\partial^{3}u}{\partial y^{3}}-\frac{\partial u}{\partial y}\frac{\partial^{2}u}{\partial x\partial y}+\frac{\partial u}{\partial x}\frac{\partial^{2}u}{\partial y^{2}}\right).$$ (2)

Note that *u* and *v* represent the flow velocities in the horizontal and vertical directions respectively while *ν*(= *μ* / *ρ*_*f*_), *μ*, *ρ*_*f*_ and *k*_0_ = −*α*_1_ / *ρ*_*f*_ denote kinematic viscosity, dynamic viscosity, density of base liquid and elastic parameter respectively. Here (*k*_0_ > 0) depicts elastico-viscous fluid, (*k*_0_ < 0) demonstrates second grade fluid and (*k*_0_ = 0) corresponds to Newtonian fluid. The Cattaneo-Christov double diffusion theory has been introduced in characterizing thermal and concentration diffusions with heat and mass fluxes relaxations respectively. Then the frame indifferent generalization regarding Fourier's law and Fick's law (which is named as Cattaneo-Christov anomalous diffusion expressions) are derived as follows:

$$q+\lambda_{E}\left(\frac{\partial q}{\partial t}+V.\nabla q-q.\nabla V+\left(\nabla .V\right)q\right)=-k\nabla T,$$ (3)

$$J+\lambda_{C}\left(\frac{\partial J}{\partial t}+V.\nabla J-J.\nabla V+\left(\nabla .V\right)J\right)=-D_{B}\nabla C,$$ (4)

where **q** and **J** stand for heat and mass fluxes respectively, *k* for thermal conductivity, *D*_*B*_ for Brownian diffusivity, *λ*_*E*_ and *λ*_*C*_ for relaxation time of heat and mass fluxes respectively. Classical Fourier's and Fick's laws are deduced by inserting *λ*_*E*_ = *λ*_*C*_ = 0 in Eqs (3) and (4). By considering the incompressibility condition (∇.**V** = 0) and steady flow with $\left(\frac{\partial q}{\partial t}=0\right)$ and $\left(\frac{\partial J}{\partial t}=0\right)$, Eqs (3) and (4 can be rewritten as

$$q+\lambda_{E}\left(V.\nabla q-q.\nabla V\right)=-k\nabla T,$$ (5)

$$J+\lambda_{C}\left(V.\nabla J-J.\nabla V\right)=-D_{B}\nabla C.$$ (6)

Now by taking the Brownian motion and thermophoresis effects in Eqs (5) and (6), then the two dimensional energy and concentration equations take the following forms:

$$u\frac{\partial T}{\partial x}+v\frac{\partial T}{\partial y}+\lambda_{E}\Phi_{E}=\alpha \left(\frac{\partial^{2}T}{\partial y^{2}}\right)+\frac{{\left(\rho c\right)}_{p}}{{\left(\rho c\right)}_{f}}\left(D_{B}\left(\frac{\partial T}{\partial y}\frac{\partial C}{\partial y}\right)+\frac{D_{T}}{T_{\infty }}{\left(\frac{\partial T}{\partial y}\right)}^{2}\right),$$ (7)

$$u\frac{\partial C}{\partial x}+v\frac{\partial C}{\partial y}+\lambda_{C}\Phi_{C}=D_{B}\left(\frac{\partial^{2}C}{\partial y^{2}}\right)+\frac{D_{T}}{T_{\infty }}\left(\frac{\partial^{2}T}{\partial y^{2}}\right).$$ (8)

Here one has the following prescribed conditions:

$$u=ax,\;v=0,\;T=T_{w},\;C=C_{w}\;\text{at}\;y=0,$$ (9)

$$u\to 0,\;T\to T_{\infty },\;C\to C_{\infty }\;\text{as}\;y\to \infty ,$$ (10)

where

$$\Phi_{E}=u\frac{\partial u}{\partial x}\frac{\partial T}{\partial x}+v\frac{\partial v}{\partial y}\frac{\partial T}{\partial y}+u\frac{\partial v}{\partial x}\frac{\partial T}{\partial y}+v\frac{\partial u}{\partial y}\frac{\partial T}{\partial x}+2uv\frac{\partial^{2}T}{\partial x\partial y}+u^{2}\frac{\partial^{2}T}{\partial x^{2}}+v^{2}\frac{\partial^{2}T}{\partial y^{2}},$$ (11)

and

$$\Phi_{C}=u\frac{\partial u}{\partial x}\frac{\partial C}{\partial x}+v\frac{\partial v}{\partial y}\frac{\partial C}{\partial y}+u\frac{\partial v}{\partial x}\frac{\partial C}{\partial y}+v\frac{\partial u}{\partial y}\frac{\partial C}{\partial x}+2uv\frac{\partial^{2}C}{\partial x\partial y}+u^{2}\frac{\partial^{2}C}{\partial x^{2}}+v^{2}\frac{\partial^{2}C}{\partial y^{2}},$$ (12)

in which *α* = *k*/(*ρc*)_*f*_, (*ρc*)_*f*_ and (*ρc*)_*p*_ stand for thermal diffusivity, heat capacity of liquid and effective heat capacity of nanoparticles respectively, *D*_*B*_ for Brownian diffusivity, *C* for concentration, *D*_*T*_ for thermophoretic diffusion coefficient, *T*_*w*_ and *C*_*w*_ for constant surface temperature and concentration respectively and *T*_∞_ and *C*_∞_ represent the ambient fluid temperature and concentration respectively. Selecting

$$\begin{array}{c} u=axf^{\prime }(\zeta ),\;v=-{\left(a\nu \right)}^{1/2}\;f(\zeta ),\;\zeta ={\left(\frac{a}{\nu }\right)}^{1/2}\;y,\\ \theta (\zeta )=\frac{T-T_{\infty }}{T_{w}-T_{\infty }},\;\phi (\zeta )=\frac{C-C_{\infty }}{C_{w}-C_{\infty }}, \end{array}$$ (13)

Eq (1) is identically verified and Eqs (2) and (7)–(12) have been reduced to

$$f^{\prime \prime \prime }+ff^{\prime \prime }-{\left(f^{\prime }\right)}^{2}-k^{*}\left(2f^{\prime }f^{\prime \prime \prime }-{\left(f^{\prime \prime }\right)}^{2}-ff^{iv}\right)=0,$$ (14)

$$\frac{1}{\mathrm{Pr}}\theta^{\prime \prime }+N_{b}\theta^{\prime }\phi^{\prime }+N_{t}{\left(\theta^{\prime }\right)}^{2}+f\theta^{\prime }-\delta_{e}\left(ff^{\prime }\theta^{\prime }+f^{2}\theta^{\prime \prime }\right)=0,$$ (15)

$$\frac{1}{Sc}\phi^{\prime \prime }+\frac{N_{t}}{N_{b}}\frac{1}{Sc}\theta^{\prime \prime }+f\phi^{\prime }-\delta_{c}\left(ff^{\prime }\phi^{\prime }+f^{2}\phi^{\prime \prime }\right)=0,$$ (16)

$$f=0,\;f^{\prime }=1,\;\theta =1,\;\phi =1\;\text{at}\;\zeta =0,$$ (17)

$$f^{\prime }\to 0,\;\theta \to 0,\;\phi \to 0\;\text{as}\;\zeta \to \infty ,$$ (18)

where (k^*^) stands for viscoelastic parameter, (Pr) for Prandtl number, (*N*_*b*_) for Brownian motion parameter, (*N*_*t*_) for thermophoresis parameter, (*δ*_*e*_) for thermal relaxation parameter, (*Sc*) for Schmidt number and (*δ*_*c*_) for concentration relaxation parameter. It is examined that (k^*^ > 0) shows elastico-viscous material and (k^*^ < 0) represents second grade material. These parameters can be specified by using the definitions given below:

$$\begin{array}{c} k^{*}=-\frac{k_{0}a}{\nu },\;\mathrm{Pr}=\frac{\nu }{\alpha },\;\delta_{e}=a\lambda_{E},\;\delta_{c}=a\lambda_{C},\\ N_{b}=\frac{{\left(\rho c\right)}_{p}D_{B}\left(C_{w}-C_{\infty }\right)}{{\left(\rho c\right)}_{f}\nu },\;N_{t}=\frac{{\left(\rho c\right)}_{p}D_{T}\left(T_{w}-T_{\infty }\right)}{{\left(\rho c\right)}_{f}\nu T_{\infty }},\;Sc=\frac{\nu }{D_{B}}. \end{array}\}$$ (19)

Skin friction coefficient is given by

$$C_{f}=\frac{{\tau_{w}|}_{y=0}}{\rho_{f}u_{w}^{2}}=\frac{{\left(\nu \frac{\partial u}{\partial y}-k_{0}\left(u\frac{\partial^{2}u}{\partial x\partial y}-2\frac{\partial u}{\partial y}\frac{\partial v}{\partial y}+v\frac{\partial^{2}u}{\partial y^{2}}\right)\right)}_{y=0}}{u_{w}^{2}},$$ (20)

The dimensionless form of skin friction coefficient is stated below:

$$Re_{x}^{1/2}C_{f}=\left(1-3k^{*}\right)f^{\prime \prime }(0),$$ (21)

where local Reynolds number is denoted by Re_*x*_ = *u*_*w*_*x*/*ν*.

### 3. Solutions by HAM

The appropriate initial approximations and auxiliary linear operators are defined as follows:

$$f_{0}(\zeta )=1-e^{-\zeta },\;\theta_{0}(\zeta )=e^{-\zeta },\;\phi_{0}(\eta )=e^{-\zeta },$$ (22)

$$L_{f}=\frac{d^{3}f}{d\zeta^{3}}-\frac{df}{d\zeta },\;L_{\theta }=\frac{d^{2}\theta }{d\zeta^{2}}-\theta ,\;L_{\phi }=\frac{d^{2}\phi }{d\zeta^{2}}-\phi .$$ (23)

The above linear operators have the characteristics

$$L_{f}\left[B_{1}^{*}+B_{2}^{*}e^{\zeta }+B_{3}^{*}e^{-\zeta }\right]=0,\;L_{\theta }\left[B_{4}^{*}e^{\zeta }+B_{5}^{*}e^{-\zeta }\right]=0,\;L_{\phi }\left[B_{6}^{*}e^{\zeta }+B_{7}^{*}e^{-\zeta }\right]=0,$$ (24)

where $B_{j}^{*}$ (*j* = 1–7) elucidate the arbitrary constants. Deformation problems at zeroth-order are

$$(1-þ)L_{f}\left[\tilde{f}(\zeta ,þ)-f_{0}(\zeta )\right]=þ\hbar_{f}N_{f}[\tilde{f}(\zeta ,þ)],$$ (25)

$$(1-þ)L_{\theta }\left[\tilde{\theta }(\zeta ,þ)-\theta_{0}(\zeta )\right]=þ\hbar_{\theta }N_{\theta }[\tilde{f}(\zeta ,þ),\tilde{\theta }(\zeta ,þ),\tilde{\phi }(\zeta ,þ)],$$ (26)

$$(1-þ)L_{\phi }\left[\tilde{\phi }(\zeta ,þ)-\phi_{0}(\zeta )\right]=þ\hbar_{\phi }N_{\phi }[\tilde{f}(\zeta ,þ),\tilde{\theta }(\zeta ,þ),\tilde{\phi }(\zeta ,þ)],$$ (27)

$$\begin{array}{c} \tilde{f}(0,þ)=0,\;{\tilde{f}}^{\prime }(0,þ)=1,\;{\tilde{f}}^{\prime }(\infty ,þ)=0,\;\tilde{\theta }(0,þ)=1,\\ \tilde{\theta }(\infty ,þ)=0,\;\tilde{\phi }(0,þ)=1,\;\tilde{\phi }(\infty ,þ)=0, \end{array}\}$$ (28)

$$N_{f}\left[\tilde{f}(\zeta ;þ)\right]=\frac{\partial^{3}\tilde{f}}{\partial \zeta^{3}}+\tilde{f}\frac{\partial^{2}\tilde{f}}{\partial \zeta^{2}}-{\left(\frac{\partial \tilde{f}}{\partial \zeta }\right)}^{2}-k^{*}\left(2\frac{\partial \tilde{f}}{\partial \zeta }\frac{\partial^{3}\tilde{f}}{\partial \zeta^{3}}-{\left(\frac{\partial^{2}\tilde{f}}{\partial \zeta^{2}}\right)}^{2}-\tilde{f}\frac{\partial^{4}\tilde{f}}{\partial \zeta^{4}}\right),$$ (29)

$$N_{\theta }\left[\tilde{f}(\zeta ;þ),\tilde{\theta }(\zeta ,þ),\tilde{\phi }(\zeta ,þ)\right]=\frac{1}{\mathrm{Pr}}\frac{\partial^{2}\tilde{\theta }}{\partial \zeta^{2}}+N_{b}\frac{\partial \tilde{\theta }}{\partial \zeta }\frac{\partial \tilde{\phi }}{\partial \zeta }+N_{t}{\left(\frac{\partial \tilde{\theta }}{\partial \zeta }\right)}^{2}+\tilde{f}\frac{\partial \tilde{\theta }}{\partial \zeta }-\delta_{e}\left(\tilde{f}\frac{\partial \tilde{f}}{\partial \zeta }\frac{\partial \tilde{\theta }}{\partial \zeta }+{\tilde{f}}^{2}\frac{\partial^{2}\tilde{\theta }}{\partial \zeta^{2}}\right),$$ (30)

$$N_{\phi }\left[\tilde{f}(\zeta ;þ),\tilde{\theta }(\zeta ,þ),\tilde{\phi }(\zeta ,þ)\right]=\frac{1}{Sc}\frac{\partial^{2}\tilde{\phi }}{\partial \zeta^{2}}+\frac{N_{t}}{N_{b}}\frac{1}{Sc}\frac{\partial^{2}\tilde{\theta }}{\partial \zeta^{2}}+\tilde{f}\frac{\partial \tilde{\phi }}{\partial \zeta }-\delta_{c}\left(\tilde{f}\frac{\partial \tilde{f}}{\partial \zeta }\frac{\partial \tilde{\phi }}{\partial \zeta }+{\tilde{f}}^{2}\frac{\partial^{2}\tilde{\phi }}{\partial \zeta^{2}}\right).$$ (31)

Here þ∈[0,1] stands for embedding parameter, ℏ_*f*_, ℏ_*θ*_ and ℏ_*ϕ*_ for nonzero auxiliary parameters and **N**_*f*_, **N**_*θ*_ and **N**_*ϕ*_ for nonlinear operators. For þ = 0 and þ = 1 one obtains

$$\tilde{f}(\zeta ;0)=f_{0}(\zeta ),\;\tilde{f}(\zeta ;1)=f(\zeta ),$$ (32)

$$\tilde{\theta }(\zeta ,0)=\theta_{0}(\zeta ),\;\tilde{\theta }(\zeta ,1)=\theta (\zeta ),$$ (33)

$$\tilde{\phi }(\zeta ,0)=\phi_{0}(\zeta ),\;\tilde{\phi }(\zeta ,1)=\phi (\zeta ).$$ (34)

When þ changes from 0 to 1 then $\tilde{f}$(*ζ*;þ), $\tilde{\theta }$(*ζ*,þ) and $\tilde{\phi }$(*ζ*,þ) display alteration from initial approximations *f*_0_(*ζ*), *θ*_0_(*ζ*) and *ϕ*_0_(*ζ*) to final ultimate solutions *f*(*ζ*), *θ*(*ζ*) and *ϕ*(*ζ*). The following expressions are obtained via Taylor's series expansion:

$$\tilde{f}(\zeta ;þ)=f_{0}(\zeta )+\overset{\infty }{\underset{\tilde{m}=1}{\sum }}f_{\tilde{m}}(\zeta ){þ}^{\tilde{m}},\;f_{\tilde{m}}(\zeta )={\frac{1}{\tilde{m}!}\frac{\partial^{\tilde{m}}\tilde{f}(\zeta ;þ)}{\partial {þ}^{\tilde{m}}}|}_{þ=0},$$ (35)

$$\tilde{\theta }(\zeta ,þ)=\theta_{0}(\zeta )+\overset{\infty }{\underset{\tilde{m}=1}{\sum }}\theta_{\tilde{m}}(\zeta ){þ}^{\tilde{m}},\;\theta_{\tilde{m}}(\zeta )={\frac{1}{\tilde{m}!}\frac{\partial^{\tilde{m}}\tilde{\theta }(\zeta ,þ)}{\partial {þ}^{\tilde{m}}}|}_{þ=0},$$ (36)

$$\tilde{\phi }(\zeta ,þ)=\phi_{0}(\zeta )+\overset{\infty }{\underset{\tilde{m}=1}{\sum }}\phi_{\tilde{m}}(\zeta ){þ}^{\tilde{m}},\;\phi_{\tilde{m}}(\zeta )={\frac{1}{\tilde{m}!}\frac{\partial^{\tilde{m}}\tilde{\phi }(\zeta ,þ)}{\partial {þ}^{\tilde{m}}}|}_{þ=0}.$$ (37)

The convergence of Eqs (35)–(37) is strongly based upon the appropriate choices of ℏ_*f*_, ℏ_*θ*_ and ℏ_*ϕ*_. Selecting suitable values of ℏ_*f*_, ℏ_*θ*_ and ℏ_*ϕ*_ so that Eqs (35)–(37) converge at þ = 1 then

$$f(\zeta )=f_{0}(\zeta )+\overset{\infty }{\underset{\tilde{m}=1}{\sum }}f_{\tilde{m}}(\zeta ),$$ (38)

$$\theta (\zeta )=\theta_{0}(\zeta )+\overset{\infty }{\underset{\tilde{m}=1}{\sum }}\theta_{\tilde{m}}(\zeta ),$$ (39)

$$\phi (\zeta )=\phi_{0}(\zeta )+\overset{\infty }{\underset{\tilde{m}=1}{\sum }}\phi_{\tilde{m}}(\zeta ).$$ (40)

The $\tilde{m}$th-order deformation problems are defined as follows:

$$L_{f}\left[f_{\tilde{m}}(\zeta )-\chi_{\tilde{m}}f_{\tilde{m}-1}(\zeta )\right]=\hbar_{f}{\hat{R}}_{f}^{\tilde{m}}(\zeta ),$$ (41)

$$L_{\theta }\left[\theta_{\tilde{m}}(\zeta )-\chi_{\tilde{m}}\theta_{\tilde{m}-1}(\zeta )\right]=\hbar_{\theta }{\hat{R}}_{\theta }^{\tilde{m}}(\zeta ),$$ (42)

$$L_{\phi }\left[\phi_{\tilde{m}}(\zeta )-\chi_{\tilde{m}}\phi_{\tilde{m}-1}(\zeta )\right]=\hbar_{\phi }{\hat{R}}_{\phi }^{\tilde{m}}(\zeta ),$$ (43)

$$f_{\tilde{m}}(0)=f_{\tilde{m}}^{\prime }(0)=f_{\tilde{m}}^{\prime }(\infty )=0,\;\theta_{\tilde{m}}(0)=\theta_{\tilde{m}}(\infty )=0,\;\phi_{\tilde{m}}(0)=\phi_{\tilde{m}}(\infty )=0,$$ (44)

$$\begin{array}{c} {\hat{R}}_{f}^{\tilde{m}}(\zeta )=f_{\tilde{m}-1}^{\prime \prime \prime }+\sum_{k=0}^{\tilde{m}-1}f_{\tilde{m}-1-k}f_{k}^{\prime \prime }-\sum_{k=0}^{\tilde{m}-1}f_{\tilde{m}-1-k}^{\prime }f_{k}^{\prime }-2k^{*}\sum_{k=0}^{\tilde{m}-1}f_{\tilde{m}-1-k}^{\prime }f_{k}^{\prime \prime \prime }\\ +k^{*}\sum_{k=0}^{\tilde{m}-1}f_{\tilde{m}-1-k}^{\prime \prime }f_{k}^{\prime \prime }+k^{*}\sum_{k=0}^{\tilde{m}-1}f_{\tilde{m}-1-k}f_{k}^{iv}, \end{array}$$ (45)

$$\begin{array}{c} {\hat{R}}_{\theta }^{\tilde{m}}(\zeta )=\frac{1}{\mathrm{Pr}}\theta_{\tilde{m}-1}^{\prime \prime }+N_{b}\sum_{k=0}^{\tilde{m}-1}\theta_{\tilde{m}-1-k}^{\prime }\phi_{k}^{\prime }+N_{t}\sum_{k=0}^{\tilde{m}-1}\theta_{\tilde{m}-1-k}^{\prime }\theta_{k}^{\prime }+\sum_{k=0}^{\tilde{m}-1}f_{\tilde{m}-1-k}\theta_{k}^{\prime }\\ -\delta_{e}\sum_{k=0}^{\tilde{m}-1}f_{\tilde{m}-1-k}\sum_{l=0}^{k}f_{k-l}^{\prime }\theta_{l}^{\prime }-\delta_{e}\sum_{k=0}^{\tilde{m}-1}f_{\tilde{m}-1-k}\sum_{l=0}^{k}f_{k-l}\theta_{l}^{\prime \prime }, \end{array}$$ (46)

$$\begin{array}{l} {\hat{R}}_{\phi }^{\tilde{m}}(\zeta )=\frac{1}{Sc}\phi_{\tilde{m}-1}^{\prime \prime }+\frac{N_{t}}{N_{b}}\frac{1}{Sc}\theta_{\tilde{m}-1}^{\prime \prime }+\sum_{k=0}^{\tilde{m}-1}f_{\tilde{m}-1-k}\phi_{k}^{\prime }\\ \;-\delta_{c}\sum_{k=0}^{\tilde{m}-1}f_{\tilde{m}-1-k}\sum_{l=0}^{k}f_{k-l}^{\prime }\phi_{l}^{\prime }-\delta_{c}\sum_{k=0}^{\tilde{m}-1}f_{\tilde{m}-1-k}\sum_{l=0}^{k}f_{k-l}\phi_{l}^{\prime \prime }, \end{array}$$ (47)

$$\chi_{\tilde{m}}=\{\begin{array}{c} 0,\;\tilde{m}\leq 1,\\ 1,\;\tilde{m}>1. \end{array}$$ (48)

General expressions $\left(f_{\tilde{m}},\theta_{\tilde{m}},\phi_{\tilde{m}}\right)$ for Eqs (41)–(43) in terms of special solutions $\left(f_{\tilde{m}}^{*},\theta_{\tilde{m}}^{*},\phi_{\tilde{m}}^{*}\right)$ are presented by the following expressions:

$$f_{\tilde{m}}(\zeta )=f_{\tilde{m}}^{*}(\zeta )+B_{1}^{*}+B_{2}^{*}e^{\zeta }+B_{3}^{*}e^{-\zeta },$$ (49)

$$\theta_{\tilde{m}}(\zeta )=\theta_{\tilde{m}}^{*}(\zeta )+B_{4}^{*}e^{\zeta }+B_{5}^{*}e^{-\zeta },$$ (50)

$$\phi_{\tilde{m}}(\zeta )=\phi_{\tilde{m}}^{*}(\zeta )+B_{6}^{*}e^{\zeta }+B_{7}^{*}e^{-\zeta },$$ (51)

in which the constants $B_{j}^{*}$ (*j* = 1–7) through the boundary conditions (44) are given by

$$B_{2}^{*}=B_{4}^{*}=B_{6}^{*}=0,\;B_{3}^{*}={\frac{\partial f_{\tilde{m}}^{*}(\zeta )}{\partial \zeta }|}_{\zeta =0},$$ (52)

$$B_{1}^{*}=-B_{3}^{*}-f_{\tilde{m}}^{*}(0),\;B_{5}^{*}=-\theta_{\tilde{m}}^{*}(0),\;B_{7}^{*}=-\phi_{\tilde{m}}^{*}(0).$$ (53)

## 4. Convergence Analysis

**Fig 1:**
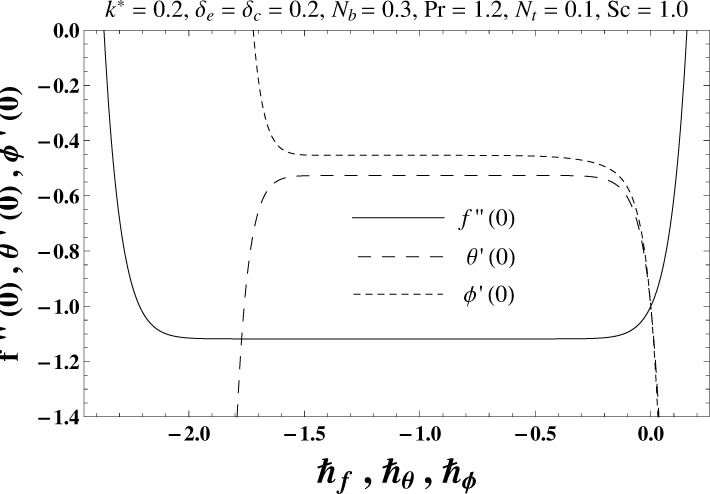
The ℏ–curves for *f*, *θ* and *ϕ* in case of elastico-viscous fluid.

**Fig 2:**
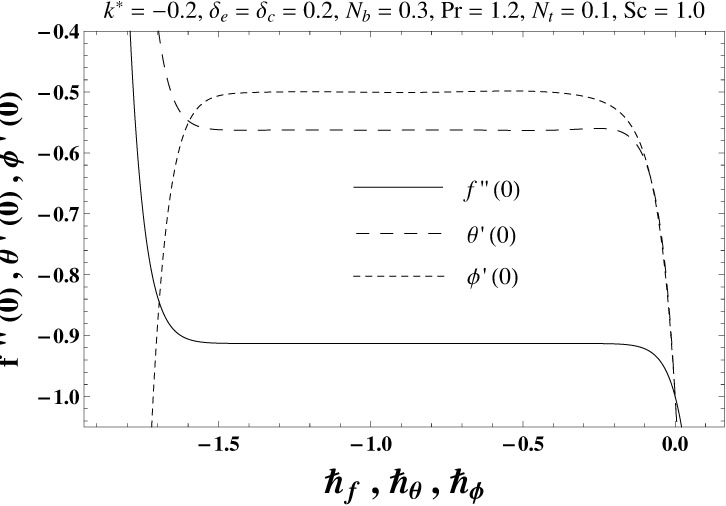
The ℏ–curves for *f*, *θ* and *ϕ* in case of second grade fluid.

Here the expressions (38)–(40) contain ℏ_*f*_, ℏ_*θ*_ and ℏ_*ϕ*_. Moreover the convergence is accelerated by the auxiliary parameters ℏ_*f*_, ℏ_*θ*_ and ℏ_*ϕ*_ in series solutions. For the purpose of determining appropriate values of ℏ_*f*_, ℏ_*θ*_ and ℏ_*ϕ*_, the ℏ–curves at 20th order of deformations are sketched to see the appropriate ranges of ℏ_*f*_, ℏ_*θ*_ and ℏ_*ϕ*_. It is apparent from Figs 1 and 2 that the admissible ranges of ℏ_*f*_, ℏ_*θ*_ and ℏ_*ϕ*_ are −1.35 ≤ ℏ_*f*_ ≤ −0.15, −1.50 ≤ ℏ_*θ*_ ≤ −0.15 and −1.60 ≤ ℏ_*ϕ*_ ≤ −0.15 for elastico-viscous fluid (k^*^ > 0) and −1.35 ≤ ℏ_*f*_ ≤ −0.15, −1.50 ≤ ℏ_*θ*_ ≤ −0.15 and −1.60 ≤ ℏ_*ϕ*_ ≤ −0.15 for second grade fluid (k^*^ < 0). Table 1 shows that the convergent series solutions of velocity, temperature and concentration fields require the 19th order of approximations for elastico-viscous fluid situation whereas the 29th order of deformations are enough for the convergent homotopy solutions of second grade material situation (see Table 2).

**Table 1:** Homotopic solutions convergence in case of elastico-viscous material for different order of deformations when k^*^ = 0.2, *δ*_*e*_ = *δ*_*c*_ = 0.2, *N*_*b*_ = 0.3, Pr = 1.2, *N*_*t*_ = 0.1 and *Sc* = 1.0.

Order of deformations	–*f*″(0)	–*θ*′(0)	–*ϕ*′(0)
1	1.10000	0.54000	0.50000
5	1.11802	0.52561	0.46919
12	1.11803	0.52643	0.45373
19	1.11803	0.52645	0.45333
25	1.11803	0.52645	0.45333
35	1.11803	0.52645	0.45333
50	1.11803	0.52645	0.45333

**Table 2:** Homotopic solutions convergence in case of second grade material for different order of deformations when k^*^ = −0.2, *δ*_*e*_ = *δ*_*c*_ = 0.2, *N*_*b*_ = 0.3, Pr = 1.2, *N*_*t*_ = 0.1 and *Sc* = 1.0.

Order of deformations	–*f*″(0)	–*θ*′(0)	–*ϕ*′(0)
1	0.90000	0.54000	0.50000
5	0.91286	0.55962	0.50144
12	0.91287	0.56209	0.50043
20	0.91287	0.56252	0.49987
29	0.91287	0.56248	0.50005
35	0.91287	0.56248	0.50005
50	0.91287	0.56248	0.50005

## 5. Discussion

**Fig 3:**
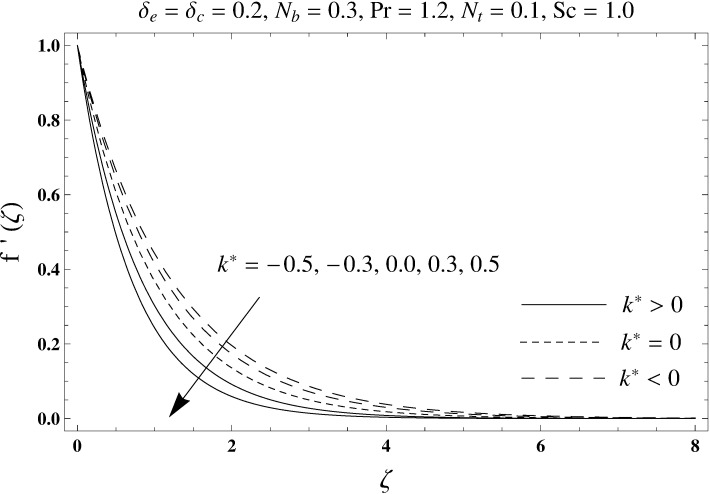
Plots of velocity profile *f*′(*ζ*) for viscoelastic parameter k^*^.

**Fig 4:**
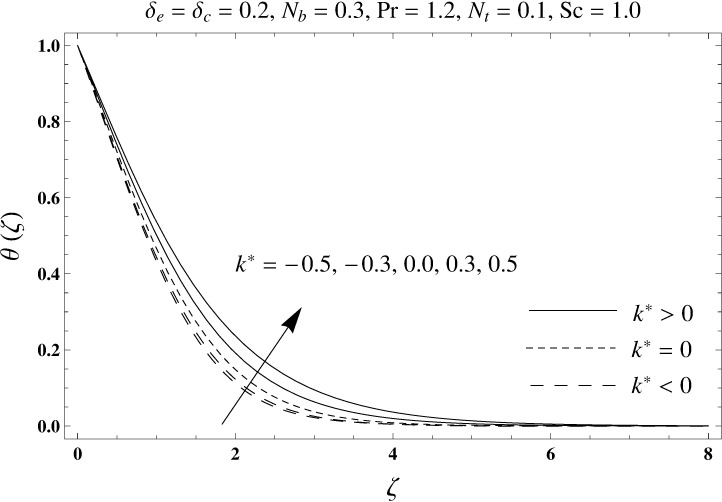
Plots of temperature profile *θ*(*ζ*) for viscoelastic parameter k^*^.

**Fig 5:**
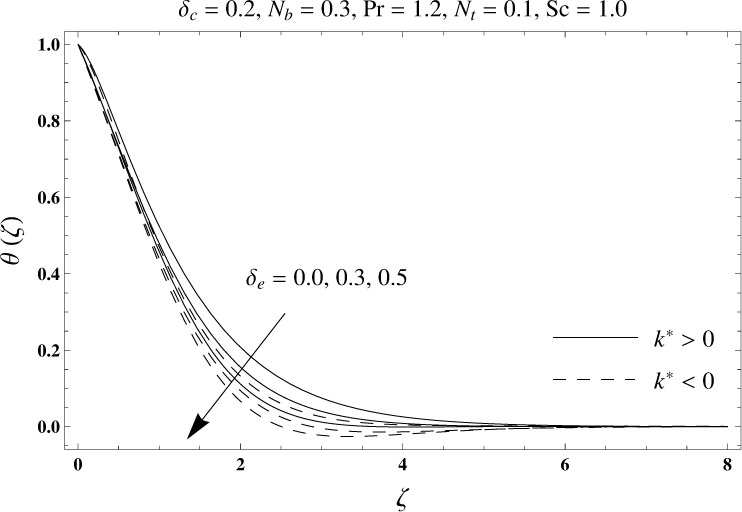
Plots of temperature profile *θ*(*ζ*) for thermal relaxation parameter *δ*_*e*_.

**Fig 6:**
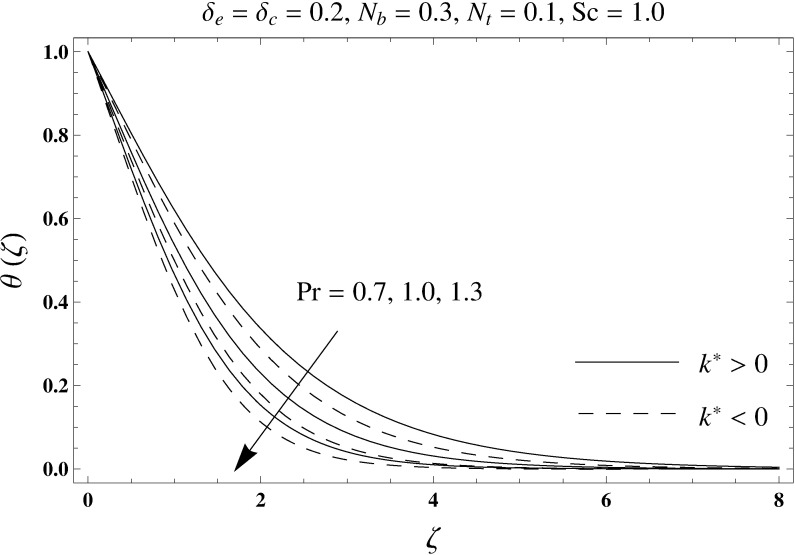
Plots of temperature profile *θ*(*ζ*) for Prandtl number Pr.

**Fig 7:**
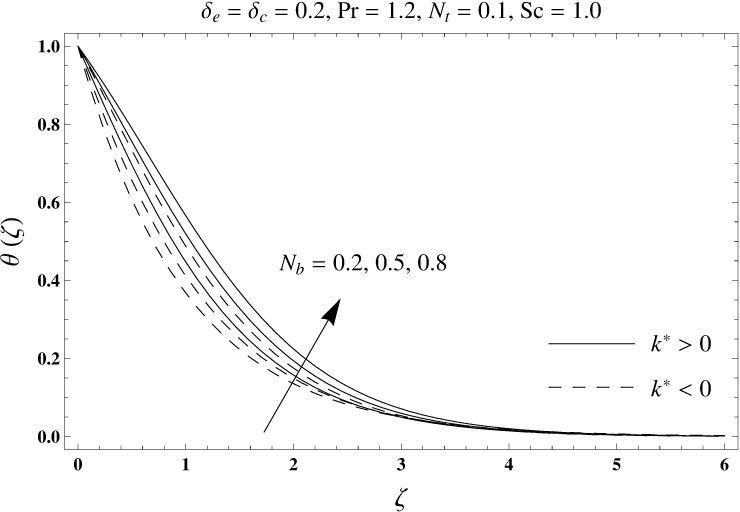
Plots of temperature profile *θ*(*ζ*) for Brownian motion parameter *N*_*b*_.

**Fig 8:**
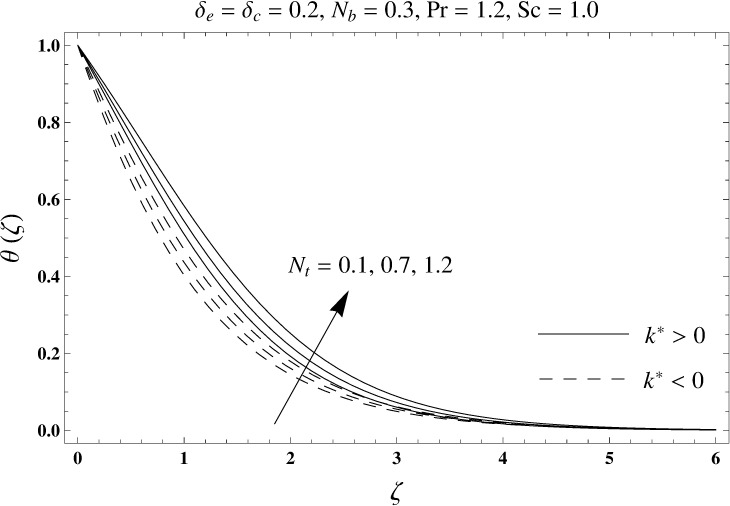
Plots of temperature profile *θ*(*ζ*) for thermophoresis parameter *N*_*t*_.

**Fig 9:**
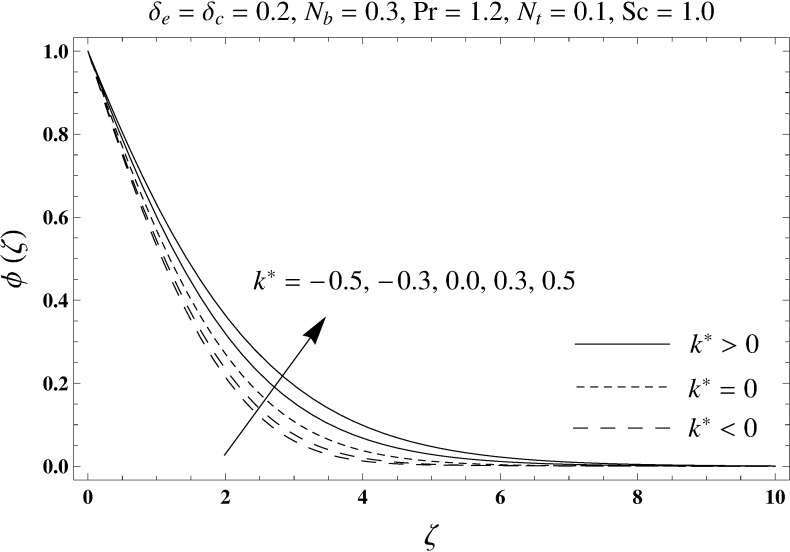
Plots of concentration profile *ϕ*(*ζ*) for viscoelastic parameter k^*^.

**Fig 10:**
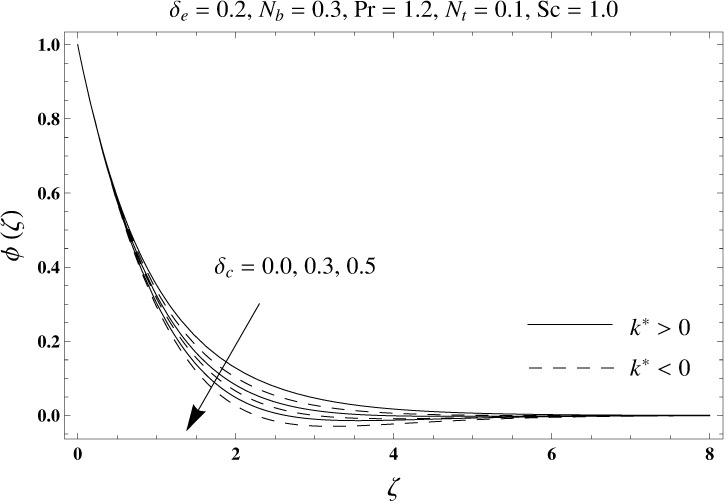
Plots of concentration profile *ϕ*(*ζ*) for concentration relaxation parameter *δ*_*c*_.

**Fig 11:**
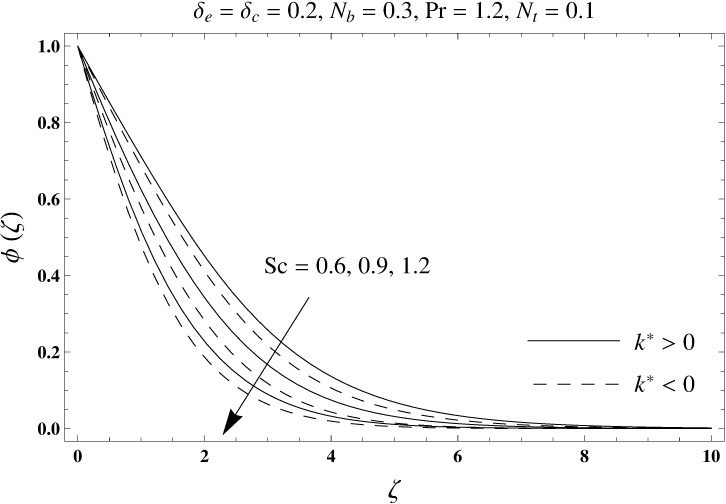
Plots of concentration profile *ϕ*(*ζ*) for Schmidt number *Sc*.

**Fig 12:**
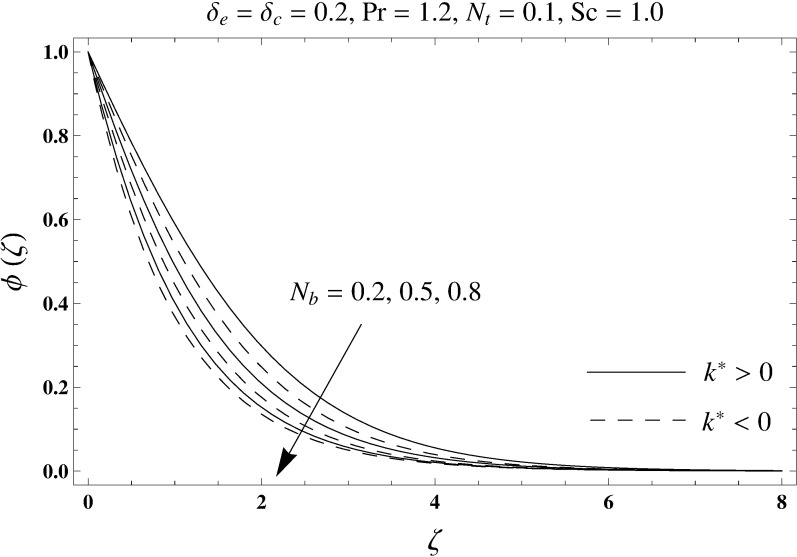
Plots of concentration profile *ϕ*(*ζ*) for Brownian motion parameter *N*_*b*_.

**Fig 13:**
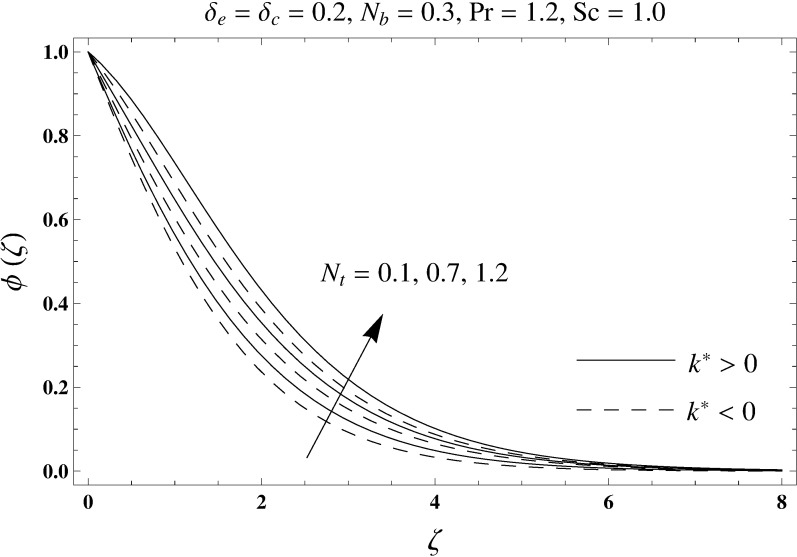
Plots of concentration profile *ϕ*(*ζ*) for thermophoresis parameter *N*_*t*_.

This portion elaborates the impacts of different interesting parameters like viscoelastic parameter (k^*^), thermal relaxation parameter (*δ*_*e*_), concentration relaxation parameter (*δ*_*c*_), Brownian motion parameter (*N*_*b*_), Prandtl number (Pr), thermophoresis parameter (*N*_*t*_) and Schmidt number (*Sc*) on the dimensionless velocity *f*′(*ζ*), temperature *θ*(*ζ*) and concentration *ϕ*(*ζ*). The results are achieved for elastico-viscous (k^*^ > 0) and second grade (k^*^ < 0) fluids respectively. Fig 3 illustrates that how viscoelastic parameter (k^*^) effects the velocity profile *f*′(*ζ*) for both fluids. From this Fig it has been analyzed that velocity field *f*′(*ζ*) decreases for the greater values of elastico-viscous parameter (k^*^ > 0) and increases for the greater values of second grade parameter (k^*^ < 0). For (k^*^ = 0), the viscous fluid flow case is recovered. The influence of viscoelastic parameter (k^*^) on temperature distribution for both fluids has been shown in Fig 4. Here the temperature *θ*(*ζ*) and thickness of thermal layer are increased for positive values of viscoelastic parameter (k^*^) while opposite behavior is analyzed for negative values of viscoelastic parameter (k^*^). Fig 5 presents the variation in the temperature distribution for different values of thermal relaxation parameter (*δ*_*e*_) for both fluids. From this Fig we can say that an increment in the values of thermal relaxation parameter (*δ*_*e*_) show decreasing behavior in temperature field *θ*(*ζ*) and thermal layer thickness. Moreover the temperature field *θ*(*ζ*) is weaker for second grade parameter (k^*^ < 0) when compared with elastico-viscous parameter (k^*^ > 0). Here (*δ*_*e*_ = 0) represents that the present model is reduced to classical Fourier's law. Fig 6 demonstrates that how the temperature field is get effected by Prandtl number (Pr) for both fluid cases. It is observed that by enhancing Prandtl number (Pr), the temperature *θ*(*ζ*) and thermal layer thickness decreases. Physically Prandtl number depends upon the thermal diffusivity. Larger values of Prandtl number correspond to a weaker thermal diffusivity. Such weaker thermal diffusivity creates a reduction in the temperature profile and related thickness of the thermal boundary layer. Moreover the thermal boundary layer thickness is less for second grade parameter (k^*^ < 0) in comparison to the elastico-viscous parameter (k^*^ > 0). Fig 7 shows the variation in temperature profile *θ*(*ζ*) for different values of Brownian motion parameter (*N*_*b*_) for both fluids. From this Fig it has been noted that by increasing Brownian motion parameter (*N*_*b*_), an enhancement appeared in temperature *θ*(*ζ*) and its related thickness of thermal layer for both fluids. Moreover the thermal layer thickness is lower for negative values of (k^*^) when compared with positive values of (k^*^). Fig 8 is drawn to depict the influence of thermophoreis parameter (*N*_*t*_) on temperature field *θ*(*ζ*) for both fluids. Larger values of thermophoresis parameter (*N*_*t*_) constitute a higher temperature field and more thermal layer thickness. The reason behind this phenomenon is that an enhancement in thermophoresis parameter (*N*_*t*_) yields a stronger thermophoretic force which allows deeper migration of nanoparticles in the fluid which is far away from the surface forms a higher temperature field and more thickness of thermal layer for both fluids. Moreover thermal layer thickness is lower for second grade parameter (k^*^ < 0) in comparison to the elastico-viscous parameter (k^*^ > 0). Fig 9 is sketched to examine that how concentration field *ϕ*(*ζ*) get effected by viscoelastic parameter (k^*^). From this Fig the concentration field *ϕ*(*ζ*) is stronger for elastico-viscous parameter (k^*^ > 0) and weaker for second grade parameter (k^*^ < 0). Fig 10 shows how the concentration relaxation parameter (*δ*_*c*_) effects concentration field *ϕ*(*ζ*) for both fluids. By increasing concentration relaxation parameter (*δ*_*c*_), both the concentration *ϕ*(*ζ*) and thickness of concentration layer decrease. It is also noticed that concentration layer thickness is lower for second grade parameter (k^*^ < 0) when compared with the elastico-viscous parameter (k^*^ > 0). From Fig 11 we observed that the larger Schmidt number forms a decay in the concentration field *ϕ*(*ζ*) and its related thickness of concentration layer for both fluids. Physically Schmidt number is based on Brownian diffusivity. An increase in Schmidt number (*Sc*) yields a weaker Brownian diffusivity. Such weaker Brownian diffusivity corresponds to lower concentration field *ϕ*(*ζ*) for both fluids. It is also observed that concentration field *ϕ*(*ζ*) is lower for negative values of (k^*^) when compared with the positive values of (k^*^). From Fig 12 it is clearly examined that a weaker concentration field *ϕ*(*ζ*) is generated by using larger Brownian motion parameter (*N*_*b*_) for both fluids. In nanofluid flow, due to the existence of nanoparticles, the Brownian motion takes place and with the increase in Brownian motion parameter (*N*_*b*_) the Brownian motion is affected and hence the concentration layer thickness reduces. It is also examined that concentration field is less for (k^*^ < 0) in comparison to (k^*^ > 0). Fig 13 shows that the higher thermophoresis parameter (*N*_*t*_) yields a stronger concentration field *ϕ*(*ζ*) for both fluid cases. Moreover the concentration field *ϕ*(*ζ*) is weaker for (k^*^ < 0) when compared with (k^*^ > 0). Table 3 shows the comparison for different values of viscoelastic parameter k^*^ with optimal homotopy analysis method (OHAM). Table 3 presents a good agreement of HAM solution with the existing optimal homotopy analysis method (OHAM) solution in a limiting sense. Table 4 is calculated in order to investigate the numerical computations of skin friction coefficient $-Re_{x}^{1/2}C_{f}$ for several values of (k^*^). Here we noticed that the skin friction coefficient is higher in case of second grade material (k^*^ < 0) while opposite trend is noticed for elastico-viscous material (k^*^ > 0). Tables 5 and 6 show the numerical computations of heat transfer rate –*θ*′(0) for various values of thermal relaxation parameter (*δ*_*e*_) in case of elastico-viscous (k^*^ > 0) and second grade (k^*^ < 0) materials respectively. Here we noticed that the heat transfer rate –*θ*′(0) has higher values for larger (*δ*_*e*_) in both materials. It is also observed that the values of heat transfer rate –*θ*′(0) for negative values of (k^*^) are higher when compared with the positive values of (k^*^). Tables 7 and 8 represent the numerical values of mass transfer rate –*ϕ*′(0) for various values of concentration relaxation parameter (*δ*_*c*_) in cases of positive and negative values of (k^*^). From these Tables we concluded that the values of mass transfer rate –*ϕ*′(0) for (k^*^ < 0) are higher when compared with (k^*^ > 0).

**Table 3:** Comparative values of $-{Re}_{x}^{1/2}C_{f}$ for different values of viscoelastic parameter k^*^.

k^*^	$-{Re}_{x}^{1/2}C_{f}$	
	*HAM*	*OHAM* [35]
0	1.00000	1.00000
−0.25	1.56525	1.56525
−0.5	2.04124	2.04124

**Table 4:** Numerical data for skin friction coefficient $-{Re}_{x}^{1/2}C_{f}$ for various values of k^*^.

k^*^	−0.3	−0.2	−0.1	0.0	0.1	0.2	0.3
$-{Re}_{x}^{1/2}C_{f}$	1.66641	1.46059	1.23950	1.00000	0.73786	0.44721	0.11952

**Table 5:** Numerical values of heat transfer rate −*θ*′(0) in case of elastico-viscous material for different values of *δ*_*e*_ when k^*^ = 0.2, *δ*_*c*_ = 0.2, *N*_*b*_ = 0.3, Pr = 1.2, *N*_*t*_ = 0.1 and *Sc* = 1.0.

*δ*_*e*_	0.0	0.1	0.2	0.3
−*θ*′(0)	0.51271	0.51943	0.52643	0.53375

**Table 6:** Numerical values of heat transfer rate −*θ*′(0) in case of second grade material for different values of *δ*_*e*_ when k^*^ = −0.2, *δ*_*c*_ = 0.2, *N*_*b*_ = 0.3, Pr = 1.2, *N*_*t*_ = 0.1 and *Sc* = 1.0.

*δ*_*e*_	0.0	0.1	0.2	0.3
−*θ*′(0)	0.54601	0.55413	0.56243	0.57116

**Table 7:** Numerical values of mass transfer rate −*ϕ*′(0) in case of elastico-viscous material for different values of *δ*_*c*_ when k^*^ = 0.2, *δ*_*e*_ = 0.2, *N*_*b*_ = 0.3, Pr = 1.2, *N*_*t*_ = 0.1 and *Sc* = 1.0.

*δ*_*c*_	0.0	0.1	0.2	0.3
−*ϕ*′(0)	0.43849	0.44576	0.45332	0.46120

**Table 8:** Numerical values of mass transfer rate −*ϕ*′(0) in case of second grade material for different values of *δ*_*c*_ when k^*^ = −0.2, *δ*_*e*_ = 0.2, *N*_*b*_ = 0.3, Pr = 1.2, *N*_*t*_ = 0.1 and *Sc* = 1.0.

*δ*_*c*_	0.0	0.1	0.2	0.3
−*ϕ*′(0)	0.48130	0.49059	0.50005	0.51006

## 6. Conclusions

Boundary-layer flow of viscoelastic nanofluids bounded by a linear stretchable surface with Cattaneo-Christov double diffusion has been discussed. The key points of the presented study are given below:

- An enhancement in the positive values of viscoelastic parameter (k^*^) demonstrate a decreasing behavior for the velocity field *f*′(*ζ*) while opposite behavior is noted for the negative values of viscoelastic parameter (k^*^).
- Larger values of Prandtl number (Pr) show decreasing trend for temperature profile *θ*(*ζ*) and its related thickness of thermal layer.
- Both temperature field *θ*(*ζ*) and its associated thermal layer thickness are reduced for larger thermal relaxation parameter (*δ*_*e*_).
- Both temperature *θ*(*ζ*) and concentration *ϕ*(*ζ*) fields show opposite behavior for increasing values of Brownian motion parameter (*N*_*b*_).
- Higher concentration relaxation parameter (*δ*_*c*_) causes a decay in the concentration field *ϕ*(*ζ*).
- Larger thermophoresis parameter (*N*_*t*_) produces enhancement for temperature *θ*(*ζ*) and concentration *ϕ*(*ζ*) fields.
- For positive values of viscoelastic parameter (k^*^), skin friction coefficient decreases while opposite trend has been observed for the negative values of viscoelastic parameter (k^*^).
- For positive and negative values of viscoelastic parameter (k^*^), both heat and mass transfer rates are higher for larger thermal (*δ*_*e*_) and concentration (*δ*_*c*_) relaxation parameters.
- The present model corresponds to the classical Fourier's and Fick's laws when *δ*_*e*_ = *δ*_*c*_ = 0.
